# Effect of Glycerol and Sisal Nanofiber Content on the Tensile Properties of Corn Starch/Sisal Nanofiber Films

**DOI:** 10.3390/polym16131947

**Published:** 2024-07-08

**Authors:** Mailson Batista de Vilhena, Marcos Vinícius da Silva Paula, Raul Costa de Oliveira, Diego Cardoso Estumano, Bruno Marques Viegas, Emerson Cardoso Rodrigues, Emanuel Negrão Macêdo, José Antônio da Silva Souza, Edinaldo José de Sousa Cunha

**Affiliations:** 1 Graduate Program in Engineering of Natural Resources of the Amazon (PRODERNA), Federal University of Pará (UFPA), Belém 66075-110, PA, Brazil; mailson.vilhena@abaetetuba.ufpa.br (M.B.d.V.); enegrao@ufpa.br (E.N.M.); jass@ufpa.br (J.A.d.S.S.); 2 School of Materials Engineering (FEMat), Federal University of Pará (UFPA), Belém 66075-110, PA, Brazil; cunhaed@ufpa.br; 3 Institute of Exact and Naturals Sciences, Federal University of Pará (UFPA), Belém 66075-110, PA, Brazil; raul.oliveira@icen.ufpa.br; 4 College of Application (CAp), Federal University of Roraima (UFRR), Boa Vista 69300-000, RR, Brazil; 5 Simulation and Computational Biology Laboratory, High Performance Computing Center, Federal University of Pará (UFPA), Belém 66075-110, PA, Brazil; dcestumano@ufpa.br; 6 Faculty of Biotechnology, Federal University of Pará (UFPA), Belém 66075-110, PA, Brazil; bviegas@ufpa.br; 7 Faculty of Chemical Engineering, Federal University of Pará (UFPA), Belém 66075-110, PA, Brazil; ecr@ufpa.br

**Keywords:** thermoplastic starch, cellulose nanofibers, sisal, acid hydrolysis, corn starch, Rietveld refinement

## Abstract

Currently, petroleum-derived plastics are widely used despite the disadvantage of their long degradation time. Natural polymers, however, can be used as alternatives to overcome this obstacle, particularly cornstarch. The tensile properties of cornstarch films can be improved by adding plant-derived nanofibers. Sisal (*Agave sisalana*), a very common low-cost species in Brazil, can be used to obtain plant nanofibers. The goal of this study was to obtain sisal nanofibers using low concentrations of sulfuric acid to produce thermoplastic starch nanocomposite films. The films were produced by a casting technique using commercial corn starch, glycerol, and sisal nanofibers, accomplished by acid hydrolysis. The effects of glycerol and sisal nanofiber content on the tensile mechanical properties of the nanocomposites were investigated. Transmission electron microscopy findings demonstrated that the lowest concentration of sulfuric acid produced fibers with nanometric dimensions related to the concentrations used. X-ray diffraction revealed that the untreated fibers and fibers subjected to acid hydrolysis exhibited a crystallinity index of 61.06 and 84.44%, respectively. When the glycerol and nanofiber contents were 28 and 1%, respectively, the tensile stress and elongation were 8.02 MPa and 3.4%. In general, nanocomposites reinforced with sisal nanofibers showed lower tensile stress and higher elongation than matrices without nanofibers did. These results were attributed to the inefficient dispersion of the nanofibers in the polymer matrix. Our findings demonstrate the potential of corn starch nanocomposite films in the packaging industry.

## 1. Introduction

Petroleum plastics are used in various applications such as food packaging, household utensils, the automotive industry, and medical products [1]. These materials, however, have a long degradation time, which can compromise the environment [2]. New alternatives are required to reduce the use of petroleum plastics. Among these alternatives, natural polymers are widely found in nature [3]. Natural polymers, or biopolymers, are generally referred to as polymers derived from biomass, including cellulose, chitosan, lignin, starch, and polypeptides [4,5]. In addition to their wide availability, biopolymers have characteristics such as biodegradability and low cost and are environmentally friendly [6]. In the spectrum of biopolymers, starch is an alternative polymer to petroleum plastics [7].

Starch is a biodegradable polymer [8] produced by plants. It acts as an energy reserve, making it a promising material for producing biodegradable plastics because of its availability, low cost, and status as a renewable material [9]. Chemically, starch is formed by two polysaccharides, amylose and amylopectin, in different proportions depending on the starch, which may influence the mechanical properties of the material [10]. Amylose is a glucose polymer with either a linear chain or a few branches; 20–30% of starch is amylose. Amylopectin is a highly branched polymer of glucose that accounts for approximately 70–80% of starch [11].

Native starch is not a true thermoplastic and is not suitable to replace petroleum plastic because it has strong intermolecular and intramolecular bonds, increasing its melting point to a value higher than the degradation temperature, limiting its processability and applications [12]. Gelatinization of starch, however, occurs in the presence of plasticizers and high temperatures, in which the three-dimensional structures of native starch rupture. Under controlled conditions, this rupture leads to the formation of a homogeneous amorphous material known as thermoplastic starch (TPS), which is a starch-plasticized material essential for producing starch-based materials [13].

One of the main roles of plasticizers is to increase the flexibility and handling properties of films. Glycerol is the most common plasticizer (in addition to water) because of the large number of hydroxyl groups present in its structure. Glycerol is a water-soluble hydrophilic plasticizer used to overcome the brittleness of films by reducing the intermolecular forces between chains [12].

Cellulose nanofibers may have mechanical properties suitable for use with TPS. Additionally, cellulose nanofibers are materials of plant origin that are normally obtained from lignocellulosic plants [14]. Chemical treatment is the primary treatment for obtaining these materials, specifically through alkaline and acid hydrolysis treatments, which aim to remove some of the amorphous compounds present in the fibers [15].

Sisal (*Agave sisalana*) is a hydrophilic plant species found in the Brazilian Amazon. Sisal leaves are hard and erect, with flat surfaces, with fruit similar to the pineapple. Sisal fibers, however, are what interest growers. These fibers are considered efficient for polymer reinforcements. They are widely available in some countries, making them advantageous for their use in developing biocomposites [16].

Several studies have been conducted on the use of natural fibers to fabricate polymer matrix composites. Islam et al. investigated the effect of adding *Moringa Oleifera* fibers to poly(lactic acid) (PLA) to improve the mechanical properties of the composites. Extrusion, injection, and compression techniques were used to fabricate the composites, and they found increases of 33 and 44% in tensile stress in relation to PLA [17]. Akindoyo et al. observed that palm oil empty fruit bunch fibers modified with poly(dimethyl siloxane) promoted an increase in tensile strength, tensile modulus, flexural strength, and flexural modulus for composites with PLA in relation to the polymeric matrix [18]. Beg et al. reported that polyamide 6.10 composites with microcrystalline cellulose fibers treated with Exxelor VA1803 exhibited better mechanical and thermomechanical properties than composites obtained from fibers treated with Bondyram 7103 [19]. Chandrasekar et al. extracted starch, nanocellulose, and bioactive compounds from banana peel; the extracted products were used to manufacture nanocomposite films [20]. They found that the addition of nanocellulose derived from banana peel promoted a 6-fold increase in the tensile stress of the nanocomposite films. Jumaidin et al. reported an increase in tensile stress for thermoplastic cassava starch composites with 1.3 and 5% coconut grass fiber [21].

The objectives of the present study were to obtain sisal nanofibers using sulfuric acid at a concentration lower than that of conventional treatments (ranging from 60 to 65%) to produce corn starch film and to evaluate the effect of different concentrations on the behavior of the nanofibers as reinforcing agents in the mechanical performance of the material produced.

## 2. Materials and Methods

### 2.1. Materials

Commercial corn starch from Maizena^®^ (São Paulo, Brasil) was used to produce the films, and glycerol 80% from Pharmapele (Belém, Brasil) was used as a plasticizer. Sodium hypochlorite and sodium hydroxide were supplied by Ypê (Goiania, Brasil) and Dinâmica (Novo Mundo, Brasil), respectively. Sulfuric acid was supplied by Dinâmica (Novo Mundo, Brasil).

### 2.2. Preparations of Sisal Nanofibers

The sisal fibers underwent mechanical defibrillation and were ground in a Model MA048 Marconi Willey knife mill to a length of approximately 0.50 mm. Alkaline treatment and bleaching were used to remove the amorphous components from the fiber surface [22]. This removal facilitated the penetration of the acid solution during hydrolysis. For the alkaline treatment of the sisal fibers, a 5% (*w*/*v*) NaOH solution was prepared. The ground fibers were treated with a NaOH solution (5% *w*/*v*) for 1 h at 80 °C in a water bath under mechanical agitation, then filtered, neutralized with distilled water (pH of approximately 7), and dehydrated in an oven at 35 °C for 24 h. During the bleaching process, the fibers were immersed in a 1% NaClO solution (*v*/*v*), washed with distilled water, filtered under vacuum, and dehydrated in an oven with air circulation at 35 °C for 24 h. The role of acid hydrolysis is to obtain a highly crystalline cellulose. The bleached fibers were subjected to acid hydrolysis in a H_2_SO_4_ solution (50% *v*/*v*) under mechanical agitation for 1 h in a water bath at 55 °C. Then, 100 mL of distilled water was added to every 1 g of fiber to interrupt the hydrolysis reaction. The resulting suspension was subjected to successive centrifugation for 20 min at 8000 rpm (discarding the supernatant) until the pH of water was reached. The fibers were ultrasonicated for 25 min and stored in a refrigerator to prevent fungal proliferation.

### 2.3. Preparation of Thermoplastic Starch Nanocomposite Films with Sisal Nanofibers

**Figure 1:**
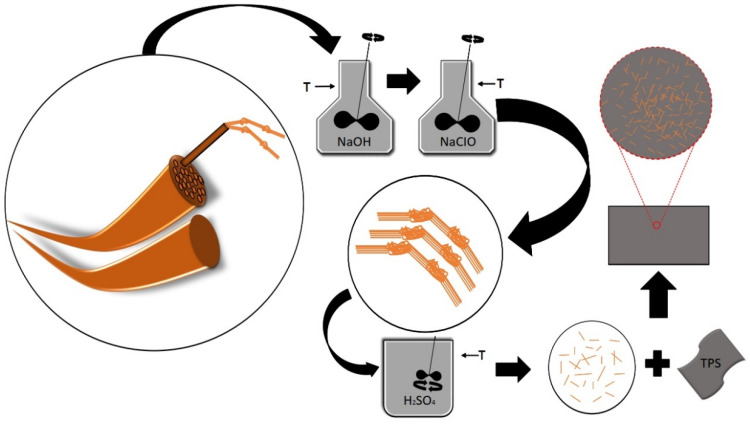
Scheme of the methodology used to obtain nanocomposite films.

The thermoplastic starch and nanocomposite films were processed using a casting technique. A film-forming solution was prepared by adding corn starch to distilled water (1:20 *w*/*v*). Glycerol was used as a plasticizing agent in concentrations of 18, 28, and 36% relative to the mass of the starch. This was subsequently homogenized and heated to approximately 85 °C until the gel point. The solution was poured into silicone molds and dehydrated in an oven at 35 °C for 24 h. The same procedure was used for the nanocomposite films, with the addition of 1 and 3% sisal nanofibers relative to starch mass. Table 1 presents the compositions of the films obtained using the solvent casting technique. Figure 1 shows a scheme for the methodology used to obtain nanocomposite films in our investigation.

### 2.4. Chemical Characterization of the Sisal Fibers

The α-cellulose, Lignin, and hemicellulose contents of sisal fibers were determined before and after the chemical treatment (alkaline and bleaching) using neutral detergent insoluble fiber and acid detergent insoluble fiber methods, as reported by Van Soest et al. [23]. For lignin, 1 g of fiber (initial mass, m_i_) was placed in a mortar with 17 mL of sulfuric acid solution (72% *v*/*v*), macerated, and stored for 24 h at room temperature. The mixture was transferred to a volumetric flask containing 3% sulfuric acid and refluxed for 4 h. Subsequently, the insoluble lignin was filtered and dried at 105 °C, and the mass of the dry material (final mass, m_f_) was determined. Lignin content was calculated using Equation (1).

(1) $$Lignin\;\left(\%\right)=\frac{m_{f}}{m_{i}}\times 100$$

For holocellulose, 3 g of fiber (initial mass, m_i_) was added to a solution containing 1 mL of acetic acid, 2.5 g of sodium hypochlorite, and 120 mL of distilled water. The mixture was then heated at 70 °C for 1 h. Then, 1 mL of acetic acid and 2.5 g of sodium chlorite were added, and the solution was heated for another hour, filtered, and neutralized. After neutralization, the material was washed three times with 10 mL of methanol and dried at 50 °C until reaching a constant mass (final mass, m_f_). The holocellulose content was calculated using Equation (2).

(2) $$Holocellulose\;\left(\%\right)=\frac{m_{f}}{m_{i}}\times 100$$

α-cellulose was determined using samples obtained from holocellulose. An initial mass of 1 g (initial mass, m_i_) was placed in 10 mL of a 17.5% (*w*/*v*) NaOH solution for 2 min. Then, another 10 mL of a NaOH solution was added and left to rest for 20 min. Subsequently, 40 mL of distilled water was added and filtered with a 50% acetic acid solution (m/v), neutralized, and dried at 105 °C until a constant mass (final mass, m_f_) was attained. The α-cellulose content was determined using Equation (3).

(3) $$\alpha \;cellulose=\frac{m_{f}}{m_{i}}\times 100$$

Hemicellulose content was calculated as the difference between the holocellulose and α-cellulose contents (Equation (4)). The determination of fiber composition was carried out in duplicate.

(4) Hemicellulose (%)=Holocellulose (%)−α celullose(%)

### 2.5. Film Thickness

The measurements were based on Nunes et al. [24], in which five random points were measured around the specimens using a digital micrometer with a resolution of 0.001 mm.

### 2.6. Scanning Electron Microscopy (SEM)

The surface morphology of the fibers before and after chemical treatment and starch films was analyzed by scanning electron microscopy using a Hitachi TM3000 scanning electron microscope operating with 5 kV beams. The samples were attached to aluminum supports using carbon tape and inserted into the SEM equipment.

### 2.7. Transmission Electron Microscopy (TEM)

The morphological characterization of the nanofiber suspension was performed using a Zeiss Leo 906 transmission electron microscope operating at an accelerating voltage of 80 kV. Before analysis, the samples were stained with a tungstophosphoric acid solution (2%) for 30 s and dehydrated with the aid of filter paper.

### 2.8. X-ray Diffraction (XRD)

X-ray diffraction analyses were performed using a D8 ADVANCE X-ray diffractometer (Bruker). The instrument was operated at a voltage of 40 kV and current of 40 mA. The acquisition time per point was 1 s, with a step of 0.02° and wavelength of CuKα1 = 1.54 Å. Crystallinity was obtained by peak deconvolution using a Gaussian function. The diffraction pattern of the sisal nanofiber was fitted by Rietveld refinement. The crystallinity index (%) of the fibers was determined using Equation (5):

(5) $$Crystallinity\;index\;\left(\%\right)=\frac{A_{c}}{A_{c}+A_{a}}\;\times \;100$$

where *A_c_* is the intensity in the crystalline region and *A_a_* is the intensity in the amorphous region.

### 2.9. Mechanical Tensile Test

The mechanical properties of T and NCs with plant nanofibers were evaluated according to the ASTM D882-02 standard [25] in a WDW 100E universal mechanical testing machine with a tensile speed of 10 mm/s and an initial distance between jaws of 50 mm. Five replicates were performed for each film type.

## 3. Results and Discussion

### 3.1. Chemical Analysis of Sisal Fibers

In Table 2, the α-cellulose, hemicellulose, and lignin contents of the sisal fibers are presented before treatment as crude fibers and after treatment by chemical alkaline and bleaching. A comparison of the results of the treated and crude fibers showed considerable increases in the percentage of cellulose and reductions in the percentages of hemicellulose and lignin. For alkaline treatment, a 5% (*w*/*v*) sodium hydroxide solution was used. Previous studies have shown that this concentration is efficient in reducing lignin and hemicellulose in natural fibers (Table 3). Higher concentrations of sodium hydroxide solution used in previous studies have shown a low influence on cellulose, hemicellulose, and lignin contents [26,27]. Our results indicated that the chemical treatments of the sisal fibers had positive effects, which was expected because the purpose of the acid hydrolysis treatment was to purify the cellulose by eliminating some of the surface materials (hemicellulose, lignin, and wax) that could interfere with the cellulose drying process.

The percentages of hemicellulose and lignin obtained in this study (Table 2) were relative to those reported by other researchers, as presented in Table 3. Teodoro et al. [28] reported hemicellulose values that were similar to those of this study. The percentages of hemicellulose and lignin showed significant differences relative to the results obtained in this study; Faruk et al. [29] emphasized that different factors, such as climatic conditions, age, and degradation, influenced both the chemical composition and structure of the fibers. The cellulose, hemicellulose, lignin, and cellulose contents extracted from five sources (wheat straw cellulose, sugarcane cellulose, cornstalk cellulose, bamboo cellulose, and rice bran cellulose) were determined after treatments with alkali, sodium hypochlorite, and acetic acid. The authors found cellulose contents close to 90% for the five celluloses, attributing this to the success of the chemical treatment used, which removed lignin, hemicellulose, and waxes from the raw fibers [30]. These results are slightly higher than ours. This chemical composition profile was also observed for sisal fibers treated with NaOH solutions in concentrations of 1, 5, and 10% [26]. Fibers from *Symphirema involucratum* stems treated with a NaOH solution also showed an increase in cellulose and a reduction in lignin and hemicellulose after chemical treatment [31]. Our results indicate the success of alkaline treatment and bleaching of sisal fibers.

**Table 3:** Cellulose (CEL), hemicellulose (HC), and lignin (LIG) contents for natural fibers before and after alkaline treatment.

Fiber	CEL_b*_ (%)	HC_b*_ (%)	LIG_b*_ (%)	CEL_a*_ (%)	HC_b*_ (%)	LIG_b*_ (%)	Ref
Sisal fiber (NaOH-5% *w/v* for 60 min)	54	25	13	78	4	9	[28]
Sisal fiber	65	12	9.9	-	-	-	[29]
Sisal fiber (NaOH-5% *w/v* for 60 min)	63.8	15.2	8.7	69.3	11.8	2.7	[26]
Borassus fruit fiber (NaOH-5% *w/v* for 30 min)	68.94	14.03	5.37	82.85	3.02	5.02	[27]
*Symphirema involucratum* stem fiber (NaOH-5% *w/v* for 60 min)	57.32	12.47	13.85	68.69	7.46	7.54	[31]
*Thespesia populnea* bark fiber (NaOH-5% *w/v* for 60 min)	70.27	12.64	16.34	76.42	9.59	12.78	[32]
Areca palm leaf stalk fiber (NaOH-5% *w/v* for 30 min)	57.49	18.34	7.26	68.54	6.13	5.87	[33]

b*—before alkaline treatment; a*—after alkaline treatment.

### 3.2. Effects of Alkaline Treatment and the Scanning Electron Microscopy of Sisal Fibers

**Figure 2:**
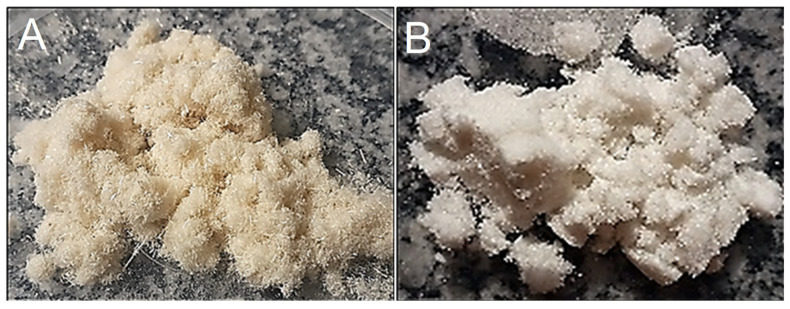
Macroscopic view of ground sisal fibers (**A**) before chemical treatment and (**B**) after chemical treatment (alkaline and bleaching).

**Figure 3:**
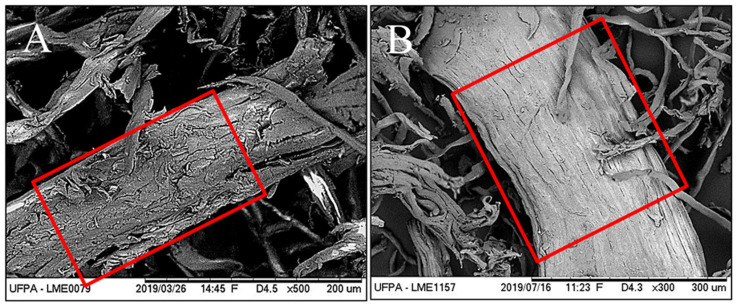
(**A**) Fibers without alkaline treatment and (**B**) with alkaline treatment.

To observe the effects of alkaline treatment on the fiber surface, comparisons were performed on macroscopic and microscopic scales, as shown in Figure 2 and Figure 3, respectively. Figure 2 illustrates the ground sisal fibers (A) treated with a sodium hydroxide solution and (B) bleached with a sodium hypochlorite solution. Discoloration was observed in relation to the fiber characteristics before treatment when observing the physical aspects of the bleached fibers. To complement and reinforce the differences resulting from the alkaline treatment, the fibers were subjected to scanning electron microscopy, resulting in successful removal of much of the material, especially lignin and hemicellulose, which were present in the fiber structure.

The morphologies of the longitudinal surfaces of the fibers before and after bleaching are shown in Figure 3. For the red demarcations, in the fibers without alkaline treatment, the bundles joined by the non-fibrous components (lignin and hemicellulose) formed microfibril structures that were less exposed. After chemical treatment, most of the components around the bundles were removed. These SEM results were corroborated by the cellulose, hemicellulose, and lignin values found for chemically treated sisal fibers (Section 3.1), which demonstrated an increase in cellulose content after alkaline treatment and bleaching. Similar results have been reported for chemically treated coconut fiber [34], *Furcraea foetida* [35], and *Bauhinia vahlii* [36].

### 3.3. SEM of TPS

**Figure 4:**
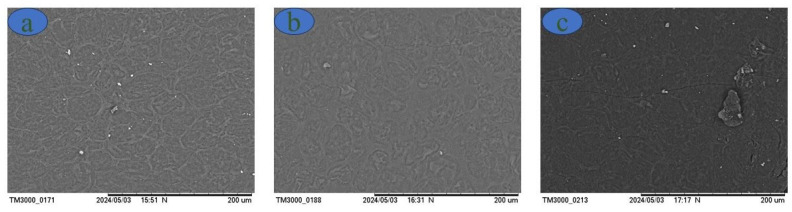
Thermoplastic films with (**a**) 18%, (**b**) 28%, and (**c**) 36% glycerol.

Scanning electron microscopy was performed on the thermoplastic starches to observe any imperfections in the formed films and to assess the influence of the percentage of plasticizer on their production. Initially, many starch granules did not melt; with increasing glycerol concentration, however, the number of starch granules decreased, as shown in Figure 4.

Figure 4 shows SEM images of the samples with concentrations of 18, 28, and 36% glycerol in relation to the starch mass. The film with the lowest glycerol content had several starch particles (white dots) that did not plasticize during the gelatinization process. With the increasing concentrations of glycerol, however, a considerable decrease in the number of these particles was observed. This phenomenon indicates an incomplete reaction in the formation of the film and the consequent influences on the mechanical performance, especially with respect to elongation, as observed in Figure 8. In other words, at lower plasticizer concentrations, large amounts of unreacted granules appeared; their presence promoted the formation of more rigid films (slightly flexible) that gained more flexibility with increasing plasticizer concentration. In addition, the presence of residual grains in films with lower glycerol content may indicate low interfacial adhesion between starch and glycerol, which may also have contributed to a decrease in tensile stress and an increase in elongation [12] (Figure 8). Similar reports have described the plasticization of arrowroot starch with 15, 30, and 45% glycerol [12]. Likewise, Khoi et al. reported that Vietnamese arrowroot starch films with 40% glycerol exhibited a surface morphology smoother than the films with 30, 20, and 10% glycerol [37]. Our SEM results indicated successful plasticization of starch with glycerol.

### 3.4. Suspension and TEM of Sisal Fibers

The suspension of sisal nanofibers was analyzed by transmission electron microscopy, and the morphological characteristics of their structures (elongated and thin) have been reported in the literature.

**Figure 5:**
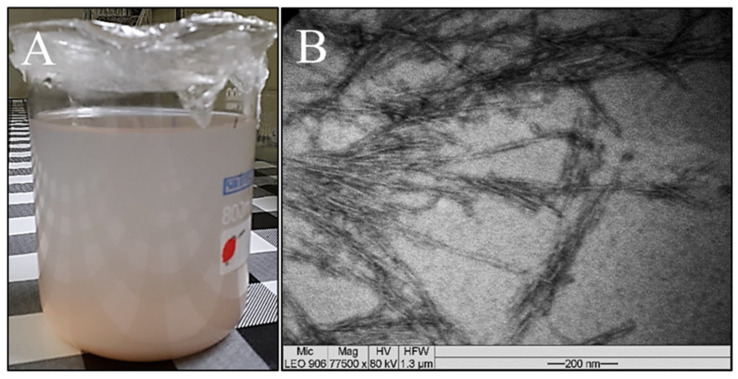
(**A**) Aqueous suspension of sisal nanofibers and (**B**) micrograph of sisal nanofibers.

Figure 5 shows the images of the suspension and the micrograph obtained by TEM from a drop of the sample. A relatively stable suspension was obtained after hydrolysis with sulfuric acid (Figure 5A). This stability may occur due to the presence of sulfate groups on the cellulose surface, which produces a negative repulsion between the nanofibers that form the stable suspension [38]. Fibers with nanometric dimensions were observed by TEM (Figure 5B). These results confirm that an acid concentration (approximately 15% less, representing a 23% reduction in acid) lower than the conventional amount, which is in the range of 64 to 65%, favored the formation of nanofibers [39]. The amorphous fraction of sisal fiber can be eliminated by acid hydrolysis where the crystalline region is preserved, resulting in the formation of nanorod-shaped nanofibers [39]. Regarding the morphology, the nanocrystals or whiskers of cellulose have elongated shapes similar to needles or rods, as portrayed by Ng et al. [40] and Adel et al. [41].

### 3.5. XRD

**Figure 6:**
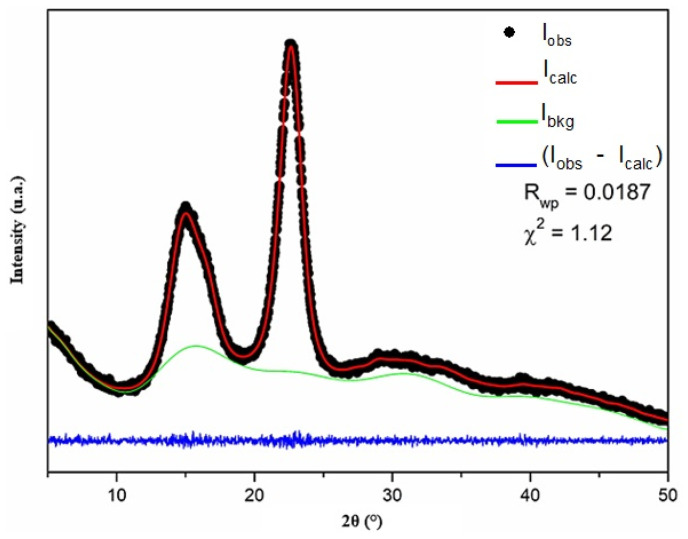
Diffraction patterns of cellulose nanofibers after Rietveld refinement.

Figure 6 shows the X-ray diffraction pattern of the cellulose nanofibers adjusted by Rietveld refinement. The crystal structure input data used for the refinement were obtained from Nishiyama et al. [42]. The reliability factors obtained after refinement were Rwp = 0.0187 and χ^2^ = 1.12 for the goodness-of-fit test. The cellulose nanofiber sample exhibited triclinic symmetry and had a P1 space group, with lattice parameters a = 10.3(2) Å, b = 6.62(6) Å, c = 5.96(6) Å, α = 79.8(5)°, β = 116.1(8)°, γ = 116.4(6)°, and volume = 328.0(5) Å^3^. According to the quality of fit calculated and based on standard data, the cellulose nanofiber sample had a type Iα crystalline structure [42]. A similar result was observed for microcrystalline cellulose [43]. On the other hand, type Iβ cellulose was observed for cellulose nanofibers from Tinwa bamboo leaves [44] and microporous cellulosic sponge observed from *Gleditsia triacanthos* pods functionalized with *Phytolacca americana* fruit extract [45].

**Figure 7:**
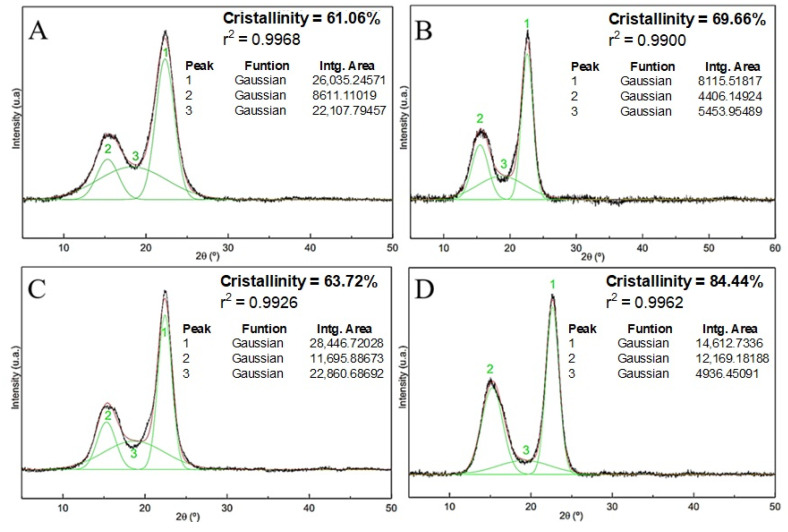
X-ray diffraction profile with deconvolution of the peaks: (**A**) fibers without treatment, (**B**) fibers with alkaline treatment, (**C**) fibers with alkaline treatment followed by bleaching, and (**D**) nanofibers.

The relative crystallinity characteristics of the fibers were calculated before they were subjected to chemical treatments in order to observe the increase in the crystallinity index after each treatment, as shown in Figure 7.

Alkaline, bleaching, and acid hydrolysis treatments were directly reflected in the increase in fiber crystallinity. Alkaline and bleaching treatments removed a large part of the surface materials with amorphous characteristics. Secondly, in the acid hydrolysis process, the disordered cellulose phase, which represents the amorphous region of the material, was destroyed, preserving its crystalline domains.

Figure 7 shows the results obtained by applying peak deconvolution to the diffraction patterns of untreated sisal fibers, sisal fibers with alkaline treatment, sisal fibers with alkaline and bleaching treatments, and nanofibers. For each analysis, the crystallinity indices (Ic), coefficients of determination (r^2^), and areas of the crystalline and amorphous peaks were highlighted. The crystallinity index of the untreated fiber (Figure 7A) was 61.06%, which is very close to the value found by Teodoro et al. [28], who obtained 60% crystallinity for sisal fibers. Ic values of approximately 60% were determined for crude fibers [36]. The crystallinity of the sisal fibers after alkaline treatment (Figure 7B) revealed that there was an increase in the crystallinity of the material relative to the raw fibers. These results reinforce the efficiency of the treatment that provides surface cleaning and removal of amorphous materials from the fibers. These results were corroborated by the IC calculation after alkaline treatment of the fibers: Curauá [46], *Coccinia grandis.* L. [47], *Acacia planifrons* bark [48], and jute [49].

Figure 7C shows that the crystallinity index for the fiber with alkaline treatment followed by bleaching was lower than that of the fiber with only alkaline treatment (Figure 7B). This fact indicates that the conditions of the bleaching performed on the sisal fibers, despite providing surface cleaning, may have affected some of the crystalline domains of the material.

The crystallinity index of the sisal nanofiber was 84.44% (Figure 7D). Teodoro et al., when using different nanofiber extraction conditions, obtained crystallinity values that ranged between 78 and 82% [28]. The increase in peak intensity at 2θ = 15.24°, which consequently increases the crystallinity of the material, reinforces the efficiencies of the conditions under which acid hydrolysis is employed, which allows high purity indices in the nanocrystals and preserves their state (cellulose Iα) [50]. Our results for Ic demonstrate the success of the chemical treatment used to obtain sisal nanofibers, which removed most of the amorphous material present in the raw fibers.

### 3.6. Film Thickness

Table 4 lists the film thicknesses and their corresponding standard deviations. The thickness of the films increased with the addition of a reinforcing agent. These results were expected because even at relatively low concentrations, materials tend to occupy spaces proportional to their volume, even those on the nanometer scale. In some situations, thicker films tend to exhibit higher tensile stresses. In these cases, there is a denser matrix and effective interaction between the matrix chains, which is an obstacle to film rupture, as observed by Galdeano et al. [51]. In our investigation, however, the range of variation in thickness is considered discrete, which is indicative of the low influence of thickness on the tensile stress tests.

### 3.7. Tensile Stress Test

In our study, we evaluated the effect of glycerol contents of 18, 28, and 36% and 1 and 3% of nanofibers on the tensile properties of nanocomposite films. Previous studies have indicated that nanofiber contents above 3% do not improve tensile properties [20]. The glycerol content generally used for starch plasticization is approximately 30% [12,14] Therefore, we decided to use glycerol amounts of 18, 28, and 36%. The mechanical tensile stress and elongation performance levels of the films with and without nanofibers and with variations in plasticizer concentration were evaluated.

**Figure 8:**
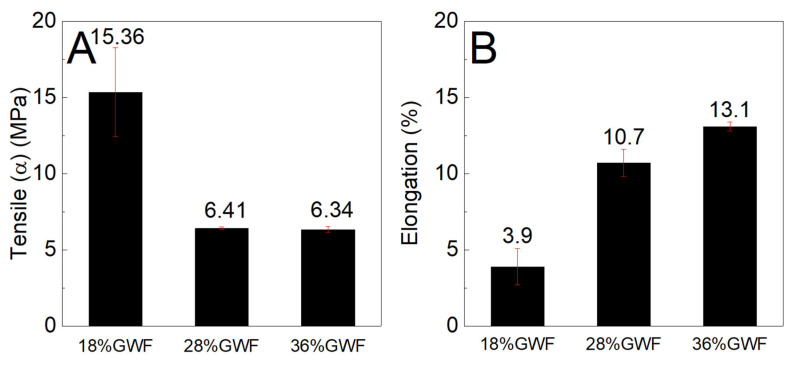
(**A**) Maximum tensile stress and (**B**) elongation characteristics of the films with 18, 28, and 36% glycerol without the use of reinforcement.

Figure 8 shows the tensile stress and elongation results of the cornstarch films with 18, 28, and 36% glycerol in relation to the dry mass of starch. The influence of the plasticizer on the mechanical results was remarkable because the film with a low glycerol concentration had a high tensile strength (approximately 15.36 MPa). With increasing concentrations of glycerol, there was a reduction in tension, indicating that the influence of glycerol content increased the reduction in mechanical strength. For 28% GWF and 36% GWF, a slight variation in tensile stress was observed. These results can be explained by the decrease in hydrogen bonding between the starch chains after the insertion of glycerol and are consistent with those reported by Vilhena et al., who reported that an increase in glycerol content decreases tensile stress because glycerol acts by relieving the hydrogen bonds that occur in starch chains [52]. In addition, glycerol forms hydrogen bonds with starch chains, thus reducing the interaction between the polymer chains. Plasticizers with a lower molar mass (such as glycerol) provide good plasticization of starch owing to the weakening of intermolecular interactions between starch macromolecules, which reduces stress tension [12]. Elongation analysis showed that elongation increased as the percentage of glycerol increased. This increase in elongation was attributed to the decrease in hydrogen bonds between the starch chains after the addition of plasticizer [37]. Similar to our results, Tarique et al. observed an increase in film elongation and a small variation in tensile stress for arrowroot starch films with 30 and 45% glycerol. This behavior after starch plasticization with glycerol is consistent with that reported by Xie et al. [53]. They found that higher levels of glycerol and the ionic liquid 1-ethyl-3-methylimidazolium acetate increased the elongation of starch films. Wang et al. also reported that the use of a higher content of polymeric ionic liquid as a starch plasticizer increased the elongation of plasticized films [54].

To compare the tensile stress and elongation results of the matrices without reinforcements, tests were performed on the films with nanofibers with concentrations of 1 and 3% in relation to starch mass. The results were compared to those of the reference matrix (film without nanofibers). Higher levels of nanofibers can reduce tensile stress owing to the agglomeration of nanomaterials, as described in an investigation conducted by Orue et al. [55].

**Figure 9:**
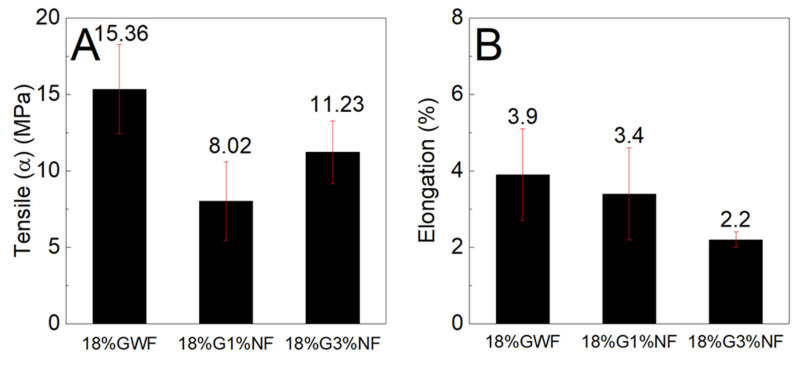
(**A**) Maximum tensile stress and (**B**) elongation characteristics of the films with 18% glycerol reinforced with 1% and 3% sisal nanofibers.

Figure 9 shows the tensile stress and elongation results of the composites reinforced with 1 and 3% sisal nanofibers in the films with 18% glycerol. The tensile stress results of the films reinforced with 1 and 3% sisal nanofibers are lower than the pure matrix 18% GWF. The figure shows that with the increase in nanofibers (from 1% to 3%) in the thermoplastic starch, there was an increase in the mechanical strength. Regarding the elongation results, there was a reduction as nanofiber content increased. These results were attributed to the poor dispersion of the nanofibers in the starch matrix, which compromised the tensile stress and elongation of the films associated to the low glycerol content.

**Figure 10:**
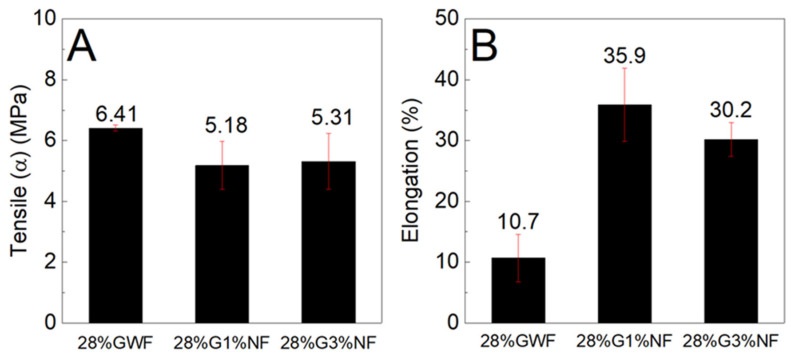
(**A**) Maximum tensile stress and (**B**) elongation characteristics of the films with 28% glycerol reinforced with 1% and 3% sisal nanofibers.

**Figure 11:**
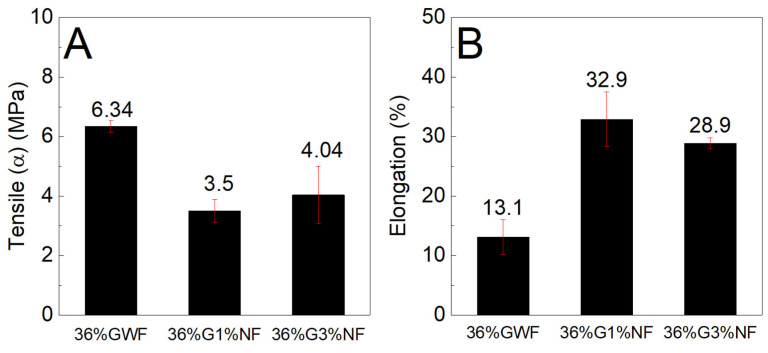
(**A**) Maximum tensile stress and (**B**) elongation characteristics of the films with 36% glycerol reinforced with 1% and 3% sisal nanofibers.

Figure 10 and Figure 11 show the tensile stress and elongation results of the composites with concentrations of 28 and 36% plasticizer (glycerol) reinforced with 1 and 3% sisal nanofibers.

Figure 10 shows that the reference matrix of 28% GWF (film without nanofibers) presents a higher tensile stress than the films reinforced with nanofibers. There were no significant differences between the tensile stress results of the films reinforced with 1 and 3% nanofibers. According to the elongation results; there was an increase of approximately 25% in the films with 1% nanofibers and an increase of 20% for the films reinforced with 3% nanofibers. High elongation values are important for the food packaging sector to facilitate the handling and transportation of packages [12].

Figure 11 shows similar findings to Figure 10 for both the tensile stress and elongation results; the nanofibers reduced the strength and increased the deformation of the films. Considering the results presented in Figure 10 and Figure 11, two hypotheses are proposed. The first hypothesis concerns the dispersion of nanofibers in the matrix, which occurred heterogeneously; this phenomenon may have contributed to the reduction in resistance. The lack compatibility between the nanofibers and thermoplastic matrix reduces the intermolecular interactions between the nanofibers and starch chains, which compromises the dispersion of the nanofibers. The poor dispersion of nanofibers in the matrix produces an inhomogeneous stress transfer, which results in a nonhomogeneous stress transfer, thus reducing the tensile stress. Moreover, the relatively long length of the nanofibers (as observed in the TEM image, Section 3.7) may increase their agglomeration and thus decrease the distribution of the nanofibers in the starch matrix, decreasing the tensile stress [5,20]. These results are consistent with those reported by Zhang et al., who stated that the addition of different sources of cellulose caused a decrease in tensile stress [30]. They stated that this behavior also occurs because of the lack of sufficient dispersion of the fillers in the starch matrix [30]. In addition, the redispersion of the nanofibers in the films was not highly efficient because, during drying, the nanofibers tended to aggregate to form rigid plates that were difficult to disperse in aqueous media; this phenomenon could have influenced the tensile stress. Our results revealed that the poor dispersion of nanofibers may have contributed to a decrease in the tensile stress of the corn starch films with sisal nanofibers. The second hypothesis concerns the increase in the deformations of the films reinforced with nanofibers in relation to the reference matrix. These increases may have occurred from the formation of a blend of the structures of starch and cellulose (which also has a polymer chain). When combined with higher concentrations of plasticizer, this phenomenon forms a material that undergoes high elongation. In contrast to our results, Tambrallimath et al. observed a different trend. By fused deposition modeling (FDM), they obtained PC-ABS ABS nanocomposites reinforced with graphene in amounts of 0.2, 0.4, 0.6, and 0.8 wt.%. Specimens obtained by FDM with 0.8% graphene exhibited an increase in tensile strength and impact resistance of 57% and 87%, respectively, compared to those of PC-ABS. These results were attributed to the alignment of the graphene and the intermolecular interactions with the polymeric matrix in association with a proper dispersion of the graphene [56]. Nimbagal et al. also reported an increase in tensile and flexural strength for a PLA-epoxy combination reinforced with 0.2 wt.% of graphene. They also described an increase in tensile flexural strength for PLA-epoxy with 0.3 wt.% of multiwalled carbon nanotubes and an increase in flexural strength for PLA-epoxy with 0.3 wt.% of multiwalled carbon nanotubes [57]. Unlike petroleum plastics, which are non-renewable and have a long degradation time, corn starch nanocomposite films are composed of natural materials that are biodegradable and renewable. Our findings suggest that there is a potential for use of cornstarch nanocomposite films in the packaging industry. Currently, a correlation between experiments and simulation is required. This correlation needs to optimize material and cost as well as material application. In this sense, Mysore et al. conducted a simulation study, where it was revealed that big sheep horn could be used for application in the outer layer of the hood of four-wheel vehicles [5]. These types of studies are ideal for finding new applications, such as ours for corn starch nanocomposite films with sisal nanofibers.

## 4. Conclusions

Sisal fibers exposed to alkaline treatment and bleaching exhibited an increase in α-cellulose content from 66.38% to 82.22%. This behavior was attributed to the removal of amorphous components from the fiber surface after alkaline treatment and bleaching. After alkaline treatment and bleaching, the sisal fibers were subjected to acid hydrolysis with 50% sulfuric acid. TEM results demonstrated that the use of 50% sulfuric acid enabled the formation of fibers with nanometric dimensions. This concentration was much lower than the most common concentrations reported in the literature (60–64%). The diffraction pattern of the sisal nanofiber sample fitted by Rietveld refinement revealed the presence of a single crystalline phase of cellulose, phase Iα. In addition, XRD results demonstrated the formation of sisal nanofibers with a crystallinity index of 84.44%. Starch nanocomposite films with glycerol and sisal nanofibers were obtained by solvent casting. When the glycerol and nanofiber contents were 28 and 1%, the tensile stress and elongation were 8.02 MPa and 3.4%, respectively. In general, the nanocomposites showed lower tensile stress and higher elongation than matrices without nanofibers. These findings were attributed to the absence of adequate dispersion of the nanofibers in the starch matrix. Our results revealed the potential of cornstarch nanocomposite films in the packaging industry.

## Figures and Tables

**Table 1:** Specification of films with different glycerol and sisal nanofiber contents.

Film Code	Film Specification
18%GWF	18% glycerol without nanofiber
28%GWF	28% glycerol without nanofiber
36%GWF	36% glycerol without nanofiber
18%G1%NF	18% glycerol with 1% nanofiber
18%G3%NF	18% glycerol with 3% nanofiber
28%G1%NF	28% glycerol with 1% nanofiber
28%G3%NF	28% glycerol with 3% nanofiber
36%G1%NF	36% glycerol with 1% nanofiber
36%G3%NF	36% glycerol with 3% nanofiber

**Table 2:** Contents of the main components of sisal fibers in their crude form and after alkaline and bleaching chemical treatments.

Sample	α-Cellulose (%)	Hemicellulose (%)	Lignin (%)
Crude Fiber	66.39 ± 0.36	26.95 ± 0.79	2.19 ± 0.58
Treated Fibers	83.72 ± 0.05	11.41 ± 0.88	0.59 ± 0.11

**Table 4:** Thickness of unreinforced corn starch films (matrices) reinforced with sisal nanofibers.

Thermoplastic Starch	Average Thickness (mm)
18%GWF	0.111 ± 0.01
18%G1%NF	0.140 ± 0.01
18%G3%NF	0.169 ± 0.02
28%GWF	0.142 ± 0.01
28%G1%NF	0.182 ± 0.03
28%G3%NF	0.185 ± 0.01
36%GWF	0.145 ± 0.004
36%G1%NF	0.182 ± 0.01
36%G3%NF	0.187 ± 0.01

## Data Availability

The original contributions presented in the study are included in the article, further inquiries can be directed to the corresponding author.
